# Ecological Momentary Assessment of emotional dysregulation and outbursts among youth with ADHD: a feasibility study of a biomarker-driven predictive algorithm in the special education pre-K and early childhood classroom settings

**DOI:** 10.3389/frcha.2025.1549220

**Published:** 2025-03-21

**Authors:** Ripudaman (Zeeba) Singh, Janav Panchal, Sami Ali, Beth Krone, Isaac J. Wert, Mark Owens, Mark Stein, Maulik V. Shah

**Affiliations:** ^1^ MaxisHealth LLC., Edison, NJ, United States; ^2^ Department of Psychiatry, Icahn School of Medicine at Mount Sinai, New York, NY, United States; ^3^ Department of Child and Adolescent Psychiatry, Grossman School of Medicine, New York University, New York, NY, United States; ^4^ Psychiatry and Pediatrics, University of Washington, Seattle, WA, United States

**Keywords:** ADHD, pre-K, emotion, emotion dysregulation, physiological sensors, children, mental health, EMA

## Abstract

**Background:**

Attention Deficit Hyperactivity Disorder (ADHD) among children younger than 6 years is quite impairing, nearly half these youth with ADHD experience school exclusion from mainstream preschool classes due to related emotional and behavioral outbursts. While a range of behavior rating scales and subjective measures are used to assess these youth, objective methods of assessment and prediction derived from technology have potential to improve therapeutic and academic interventions outcomes for these youths. We hypothesized that biometric sensors would provide objective, highly sensitive and specific information regarding the physiological status of children prior to an impulsive outburst and could be feasibly implemented using a wearable device in the special education classroom.

**Methods:**

We recruited two whole classrooms (*N* = 5 youth in the pre-K class and *N* = 5 youth from the first grade) of a specialized therapeutic day-school for youth with ADHD and other psychiatric and developmental disorders to examine feasibility of obtaining continuous physiological data associated with behavioral and emotional outbursts through smartwatch use. Children wore a sensor watch during their daily classroom activities for two weeks and trained observers collected data using behavioral logs. Using Ecological Momentary Assessment methodology, to examine correlations between objective sensor data and observer observation. Data collected from parents regarding prior night's sleep was also examined.

**Results:**

All participants completed the study. With a few tolerability or palatability issues. Associations were found between physiological and behavioral/questionnaire data. The methodology holds promise for reliably measuring behavioral and emotional outbursts in young children.

**Conclusions:**

This is the first study to use a bio-marker device *in vivo* among severely dysregulated pre-school aged youth throughout a full school day. This study established the feasibility of utilizing sensor derived physiological data as an objective biomarker of ADHD within the special education therapeutic classroom. Further research with larger samples is required to build a more robust and personalized AI predictive model.

## Introduction

The prevalence of attention-deficit/hyperactivity disorder (ADHD) is estimated to be about 2% in preschool aged children (1). The prominent symptoms of over-activity and impulsivity in preschool ADHD are associated with impairments in cognition, executive functioning, and emotional dysregulation (2). Emotional dysregulation occurs in approximately 25%–45% of these preschoolers with ADHD (3, 4). This number rises by early elementary school, where approximately 48%–54% of children with ADHD display emotion dysregulation (4). More-so than with other symptoms, emotional dysregulation in preschool results in school expulsions (1), and many parents struggle to find a suitable classroom setting for their children (1). School exclusions are often considered necessary for these youth but exacerbate the course of their impairments as high rates of comorbidities, including specific learning disabilities that include speech and language disorders, and cognitive problems are not adequately addressed. Many youths with ADHD and emotional dysregulation require smaller classroom settings with applied behavioral strategies in class, and with extensive teacher support. Even within these smaller class settings, with dedicated and well-trained observers, emotional outbursts are often difficult to anticipate, and these children remain difficult to manage. Wearable technologies offer the benefit of objective measurement of physiological data that may not be otherwise observable markers of emotional regulation failures, and impending outbursts.

Several studies suggest biologic underpinnings for impaired self-regulation, including electrodermal activity marking dysregulated emotion reactivity, and respiratory sinus rhythms marking deficits of emotion regulation in youth with ADHD (5). A recent study examined the feasibility and palatability of a biomarker device for use with older children with ADHD who were admitted to an in-patient hospital setting (6). However, no studies have examined their use with younger elementary school and pre-school aged children, and no studies have been conducted with these youth *in vivo* within their daily lives in a community setting. In fact, according to a recent review, since 2000, more than 45 studies examined feasibility of wearable technologies and the physiological data that they capture in increasing physical activity and reducing obesity among older youth, with only 21 being considered well-designed clinical trials (7). Only one of these was conducted among youth aged 3 years old, and one among youth ages 5–7 years old, and these both restricted assessment to accelerometer data, which has been historically used in ADHD research, and do not require direct, consistent contact with skin (7). Wearable technologies comprising multiple sensors (e.g., heart rate and accelerometers) demonstrate much greater promise for prediction and diagnosis among children with psychiatric disorders (8).

As youth with ADHD and emotional dysregulation, at risk for more severe and continuing impairments, we proposed to evaluate a multi-sensor device among pre-school-aged youth for monitoring and predicting disruptive behaviors. However, inclusion of preschool aged youth in such as study presents specific challenges, including their having smaller wrists and different symptom profiles than school-aged youth. Pre-school children are more physically active and hence generate more physiological noise in the sensor data. Their having different behavioral phenotypes might be reflected in different age-related algorithms that separate from those of older elementary school aged youth.

## Hypothesis

This study aimed to evaluate the feasibility of collecting objective physiological data alongside behavioral correlates potentially associated with emotion dysregulation in young children diagnosed with ADHD and related disorders. We hypothesize there are associations between objective physiological measures of arousal and emotional reactivity of young children in the classroom.

## Methodology

Participants were 10 children, 6 male and 4 female with a mean age of 5.6 years who attended pre-K and 1st grade classes at the League Treatment and Education Center in Brooklyn, New York., a therapeutic day-school for children with severe mental health and developmental disabilities.

Our study was approved by WCG IRB (Protocol #: 2023-0100). Approval by the League School's independent ethics board was also obtained.

In partnership with The League's leadership team, psychology and social work staff, and in accordance with the recommendations of the League's independent Ethics Panel, all students in the pre-K and 1st grade classrooms were invited to participate in the study due to the school's concern that no child be excluded or singled out. All parents accepted the invitation and provided consent for their child to participate. The protocol defined inclusion criteria for the youth were a prior or current diagnosis of ADHD, Vanderbilt (Parent or Teacher) scale score >27 for total ADHD symptoms and observed emotion regulation difficulties during the week prior to their participation in the study. These emotion regulation difficulties were observed by teachers, the study psychiatrist, and the school's behavior support team, including licensed mental health professionals. Behaviors meeting the threshold for emotion difficulties included behavioral outbursts that disrupted class time or peer activities and that were more frequent in number, intensity, and duration than expected for the child's age. Eligibility for the protocol and presence and severity of current ADHD symptoms was confirmed by the child and adolescent psychiatrist (MO).

All youth assented at the time of consent. Due to the nature of the study, youth also continued to assent daily, each time the sensor watch was placed on children's wrists.

The ethnic background of the participants on this study were as follows: 70% AA, non-Hispanic, 10% white, non-Hispanic, 10% biracial, Hispanic, 10% Asian, non-Hispanic. With respect to parenting: 50% were single parents. The remaining families included Legally Authorized Representatives (i.e., custodial grandparents), co-parents, or joint custody. Socio-economic status was low, with an average Hollingshead Scale Score (9) of 30.4 (SD = 15.5).

No children were taking stimulant medications. 30% of youth were taking Guanfacine extended release (GXR) during the study period.

The children in this study had a primary diagnosis of ADHD accompanied by emotion dysregulation and were observed for up to 20 h over a 10-day period. Students wore the watch for a total of 20 h during the study: 4 h per day for 5 days during the school day. Following this observation period, data from physiological sensors, daily behavioral logs maintained by observers, and information provided by parent and teacher-rated forms were analyzed. Each of these measures has been elaborated on below.

### Ecological Momentary Assessment measures

#### Physiological measures

The Biosensor wearable^1^ was supplied to collect data through four sensors and a gyroscope: a Ventral electrodermal activity (EDA) sensor, a 4-channel multi-wavelength photoplethysmogram (PPG) sensor, a high precision 3D microelectromechanical accelerometer and gyroscope, and a digital temperature sensor (SKT).

#### Behavioral measure

The Antecedent-Behavior-Consequence (ABC) Behavior Chart (10, 11) is a tool that assists with direct observation of youth within a classroom setting. When coded by trained observers the tool makes it easy to chart the context for disruptive behaviors. By charting the antecedents of behaviors, the behavioral response to these triggers, and the consequences immediately following the behaviors, patterns causing and maintaining disruptive behaviors can be elucidated.

### Clinical characterization measures

**The Behavior Assessment System for Children (BASC)** (12, 13) is a norm-referenced instrument used to measure the intrapersonal competencies, functional impairments, and clinical characterization across a broad range of psychopathologies among children or adolescents. It is commonly used in therapeutic educational settings for the purpose of monitoring child behavior and/or emotional status throughout development. This rating scale is suitable for ages 2 and above. The BASC was collected from student charts or completed by teachers.

**The Vanderbilt ADHD Diagnostic Rating Scale (VADRS)** (14–16) is a commonly used measure of ADHD symptoms during the past 6 months Although the VADPRS was originally developed for children ages 6–12, it is now widely used for children ages 4–17 years and as a treatment outcome measure.

**Social Communication Questionnaire (SCQ)** (17) is a caregiver-report of social communication that is commonly used in assessing Autism (ASD) symptomatology but is also effective in assessing functional communication in other disorders, such as verbal disabilities among children over the age of 4 who have a mental age of at least 2 years.

### Sleep habits

Information about sleep was captured using two common questionnaires and a sleep log that was developed for this study (see Supplementary Appendix I). Parents were asked to record naps, nap duration, bedtime, sleep onset latency, number of night awakenings, reasons for night awakenings, duration of night awakenings, wake time, total sleep duration, and sleep setting.

**Children's Sleep Habits Questionnaire (CSHQ)** (18): is a Parent-reported screening survey designed to assess behavioral and medically based sleep problems in school children, aged 4–10 years.

**Children's Sleep-Wake Scale (CSWS)** (19): is a caregiver-report measure of behavioral sleep quality in 2- to 8-year-old children.

### Data collection procedure

Participants were observed for up to 10 days, during which approximately 20 h of data were collected as they engaged in their regular school activities. Each participant was monitored by a trained observer, who reported daily to the principal investigator. Prior to assignment, observers underwent training to ensure consistency and minimize disruption to classroom activities. Observers recorded the participants’ behaviors manually, maintaining detailed ABC logs that included observer notes on the child's activities and perceived emotional status, as well as watch distribution logs, which tracked when the watch was on or off the participant's wrist. Additionally, parents were instructed to complete a daily sleep log for their child.

#### Physiological data acquisition and processing

**Figure 1:**
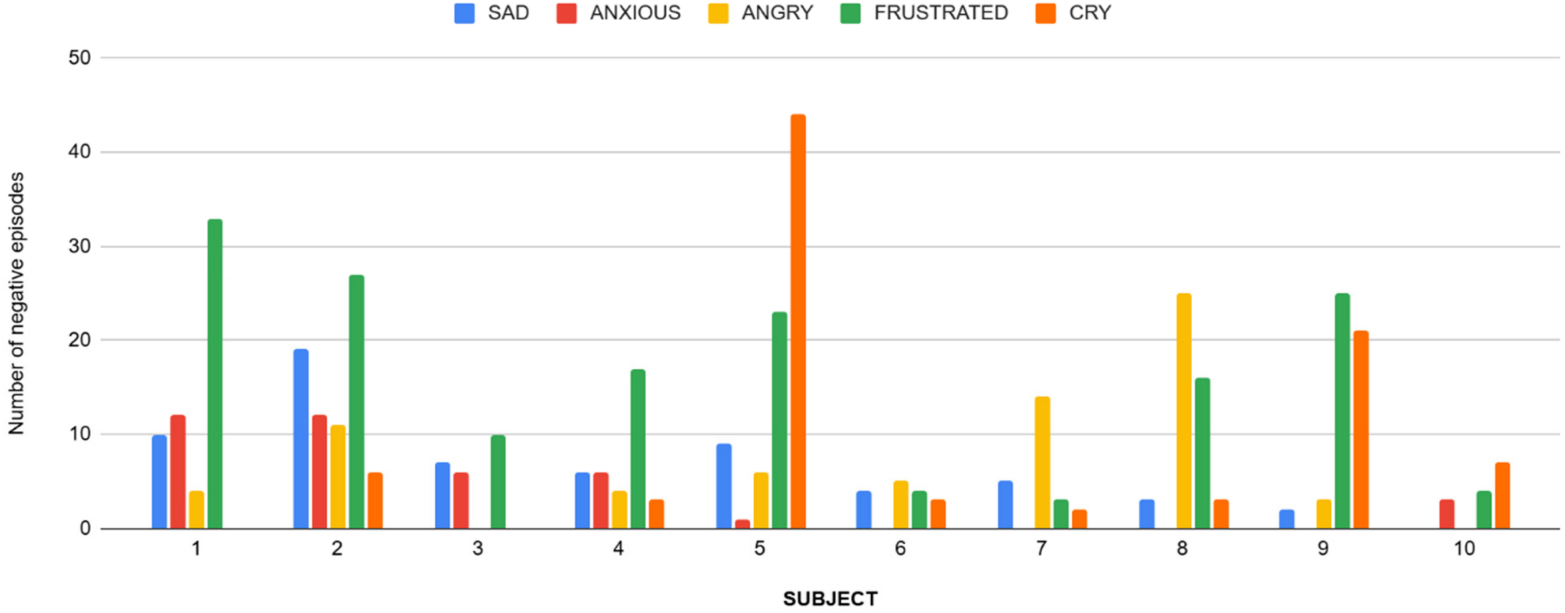
A bar chart illustrating the total number of negative emotions/abnormal events as captured via Ecological Momentary Assessment by the observers.

Data collected daily from participants was synchronized via Bluetooth to the biosensor wearable^1^ companion app. The raw data were filtered to extract specific features related to electrodermal activity (EDA), skin temperature (SKT), and accelerometer (ACC) signals. Python was utilized as the programming language for coding and processing these signals. To facilitate analysis, each signal was divided into usable segments for each subject, enabling the detection of minor changes within the signals. The following features were extracted:

• EDA: The EDA signal, provided as a filtered signal by biosensor wearable^1^, was decomposed using the cvxEDA algorithm into tonic skin conductance level (SCL) and phasic skin conductance response (SCR). Statistical features such as mean, maximum, and standard deviation were subsequently extracted.
• **SKT:** Basic descriptive statistics were computed from the skin temperature biosignal.
• **ACC:** The physical activity count, which reflects the intensity of physical activity, and the Euclidean norm minus one (ENMO) were computed. ENMO was particularly important for excluding periods of moderate to vigorous physical activity, as these periods could exhibit physiological attributes like those observed during episodes of emotion dysregulation.
• **Abnormal events statistic**: Information noted by the observers if it belonged to a negative emotion, was noted as “abnormal events” and were further statistically analyzed (Figure 1).

### Statistical analyses

Statistical analyses was done on Python 3.9. Pearson correlation was selected to evaluate the relationship between EDA, SKT, ACC, and additional features related to sleep patterns and behavior due to its suitability for continuous numerical data and its ability to measure both the magnitude and direction of linear relationships. Since physiological responses such as skin conductance (EDA), temperature changes (SKT), movement patterns (ACC), and behavioral indicators interact in complex ways, it was essential to assess how strongly these features correlate with each other. The assumption of linearity was validated through scatter plots, confirming that the relationships between these biomarkers exhibit a roughly linear structure. Unlike rank-based methods such as Spearman correlation, Pearson effectively captures the actual strength of association rather than just monotonic trends. To maintain a consistent approach across all features, both the mean and standard deviation (std) were used for feature computation. Based on these considerations, Pearson correlation was the most appropriate choice for analyzing the relationships among these physiological and behavioral features. Thus, electrodermal activity (EDA) and skin temperature (SKT) features were entered into three Pearson correlation matrices, the first was used to examine exploratory correlations with clinical characteristics, including clinical and behavioral information including physician rated diagnoses, teacher, and parent reports. The second was used to examine associations with symptom inventories including parent and teacher rated symptoms of sleep, ADHD, oppositional defiance, anxiety, and depression. The third was used to examine associations with classroom observer rated Ecological Momentary Assessment of behavior.

## Results

1. **Palatability and Safety**

The biosensor wearable^1^ watch was worn by all participants, within the classrooms, during the specified time frame.

According to the palatability survey:

• Teachers reported children initially played with the watch and were distracted by it until they acclimated to wearing it on the first day. However, the watches did not disrupt learning, and the watches lost novelty within hours.
• One child complained that the watch was too tight and uncomfortable, but this was resolved when the teacher loosened the band and data quality was maintained. Validation of the bio signals’ reliability when loosened was supported by biosensor wearable's high signal quality, the absence of errors during data collection and transfer, and the significant (non-zero) values obtained after filtration and feature extraction.
• One parent reported the watch leaving a mark on a child's wrist. The marks spontaneously resolved on the same day, and the watch was placed more loosely the next day.
• One child complained that the watch was uncomfortable and had the watch temporarily removed, but it was placed back on his wrist during the same session without complaint, and the child completed the study.

2. **Pilot Data on Associations of Physiological Measures and Behavior (See Table 1, and Figures 1–11)**

**Table 1:** List of associations between physiological features, sleep forms (CSHQ, DSF) and other scales such as (SCQ, BASC and parent/teacher vanderbilt).

Feature	Degree	Nature	Inference
SCR mean	Weak	Positive	EDA (SCR mean, std, max) features showed a weak positive association with BASC variables such as hyperactivity, aggression, externalizing, anxiety.
SCR peaks number max	Moderate	Positive	Night awakenings count from the DSF form is moderately positively associated with max number of SCR Peaks from EDA signal.
SCL std	High	Positive	SCL std (tonic EDA component) showed a high positive association with Mean temperature.
SCL max	Moderate	Negative	Average and total abnormal counts by observer are moderately negatively associated with EDA features SCL max
Temp std	Moderate	Positive	Std Temperature shares a moderate positive association with Average and total abnormal counts by observer. Night wakings, Sleep onset delay from CSHQ forms and Abnormal events count (total average) are moderately positively correlated with Temperature features (mean, std).
Temp max	High	Positive	Sleep onset latency was highly positively association with Temp max
Bed resistance	Weak	Positive	Total average count of abnormal events by observer shows a Weak positive association with Bed resistance
Sleep disordered breathing	High	Negative	Sleep disordered breathing, a sub scale from the CSHQ sleep form showed a high negative association with BASC scales—aggression and externalizing behaviors.
DSF	High	Positive/Negative (both)	The inhouse DSF (Daily sleep form) was Highly associated with an established sleep form CSHQ (Childrens Sleep Habit Questionnaire) and in a POSITIVE association for most subscales.
Night awakenings counts	High	Negative	DSF features Night awakenings count and duration are highly negatively associated with Teacher reported Vanderbilt on Inattention, Hyperactivity and Conduct scales.
Night awakenings duration			
Wake time	Moderate	Positive	Parent and teacher Vanderbilt for ODD, Teacher Vanderbilt for Hyperactivity and ODD, were moderately positively associated with Wake times from DSF.
Total sleep duration	Weak	Negative	Total count of abnormal Average count of abnormal events as observed shows a Weak negative association with Total sleep duration.
Depression BASC	Moderate	Positive	Wake time from DSF is moderately positively associated with Depression from BASC.
Somatic BASC	High	Negative	SCL mean and Temperature (std) is highly negatively associated with Somatic subscale from BASC.
Total count of abnormal by observer	High	Positive	Parent reported inattention from Vanderbilt is highly positively associated with the total count of abnormal events as noticed by the observer.
Total/Average count of abnormal by observer	Moderate	Positive	Temp std is moderately positively associated with Total average counts of abnormal events as noticed by the observer.
	Moderate	Negative	SCL max is moderately negatively associated with Total average counts of abnormal events as noticed by the observer.
	Weak	Positive	Bed resistance, Functional Communication, PVanderbilt_I Isx, Hsx—have a weak positive association with Total average abnormal events as noted by the observer.
PVanderbilt_H & C	Moderate	Positive	Sleep anxiety, Bed resistance are moderately positively associated with Parent reported Vanderbilt scales Hyperactivity and Conduct respectively
SCQ	High	Positive	CSHQ Score and other subscales like Sleep onset delay, Parasomnias, Daytime sleepiness is highly positively associated with Social communications questionnaire
DSF Descriptive results			Children's parents who were prompted to fill the Daily sleep form each night, were analyzed and reported the following: most children would not take a nap in the day. The most frequent bedtime was 8pm to 10 pm, while wake times were 6 am to 8 am. With total sleep duration around 8 h. Children were reported to most frequently fall asleep within 45 min of lying in bed.

**Figure 2:**
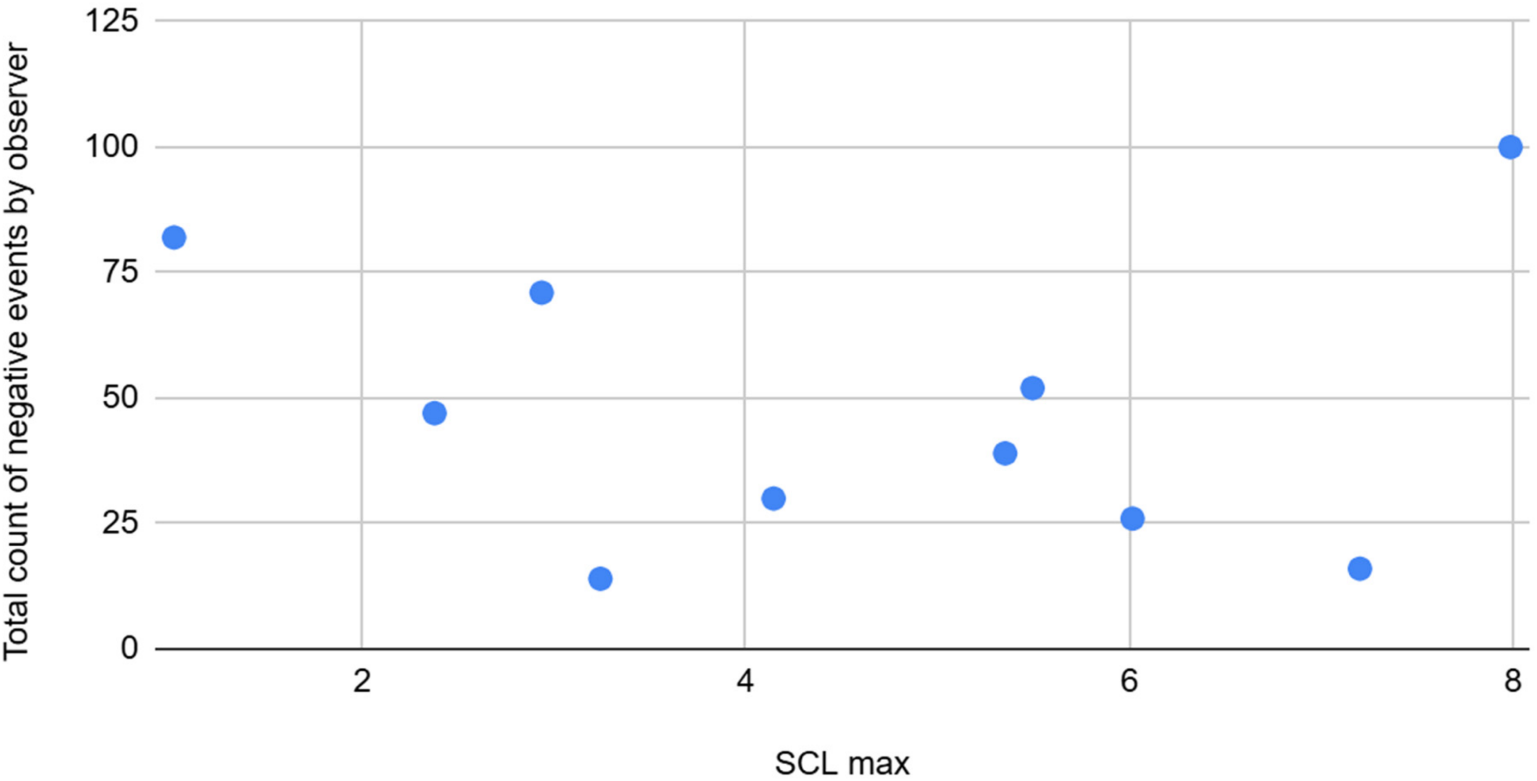
The graph depicting some of the associations between physiological/behavioral variables: SCl max vs. total count negative events by observer.

**Figure 3:**
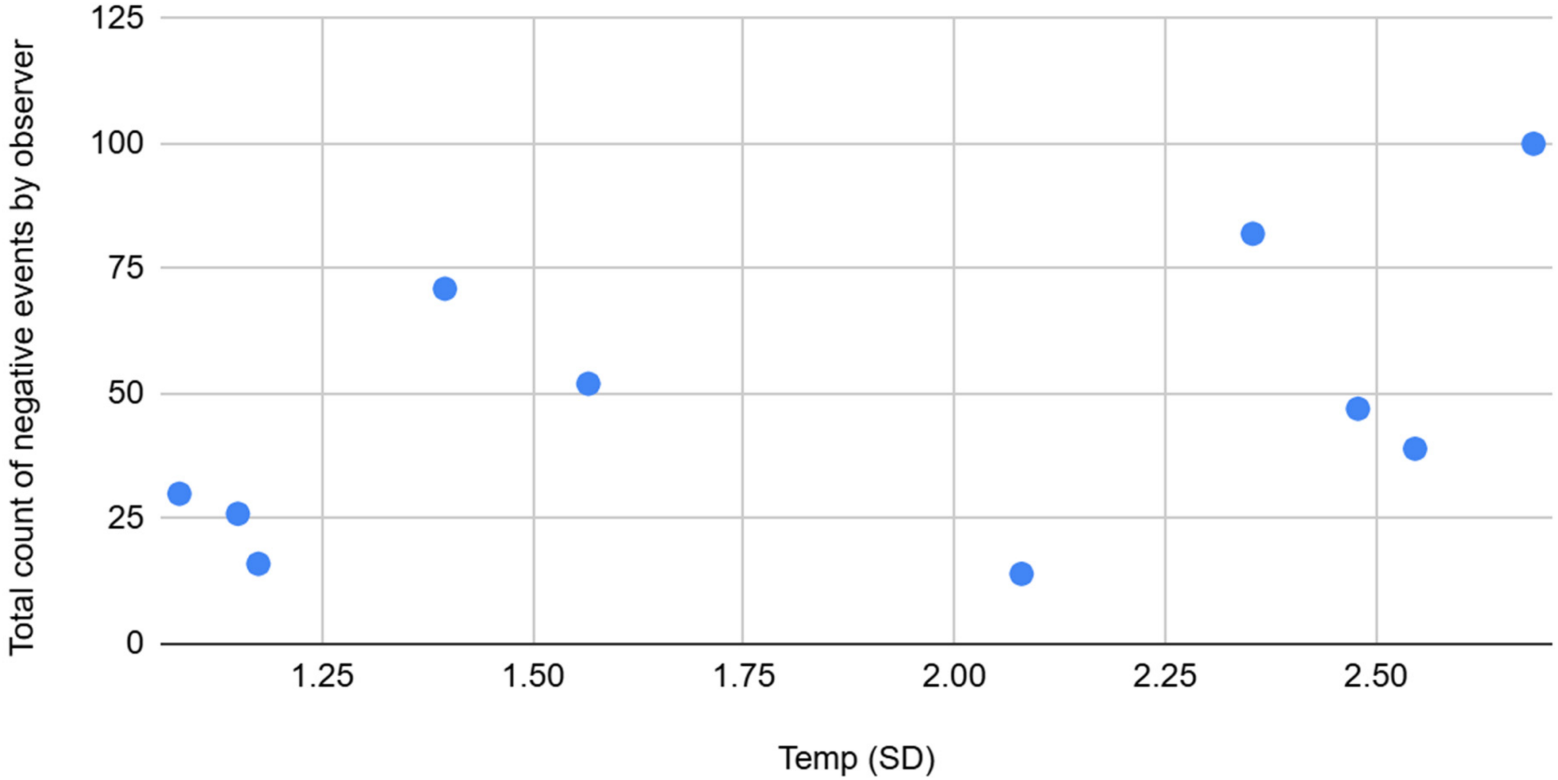
The graph depicting some of the associations between physiological/behavioral variables: Temperature (SD) vs. total count of negative event by observer.

**Figure 4:**
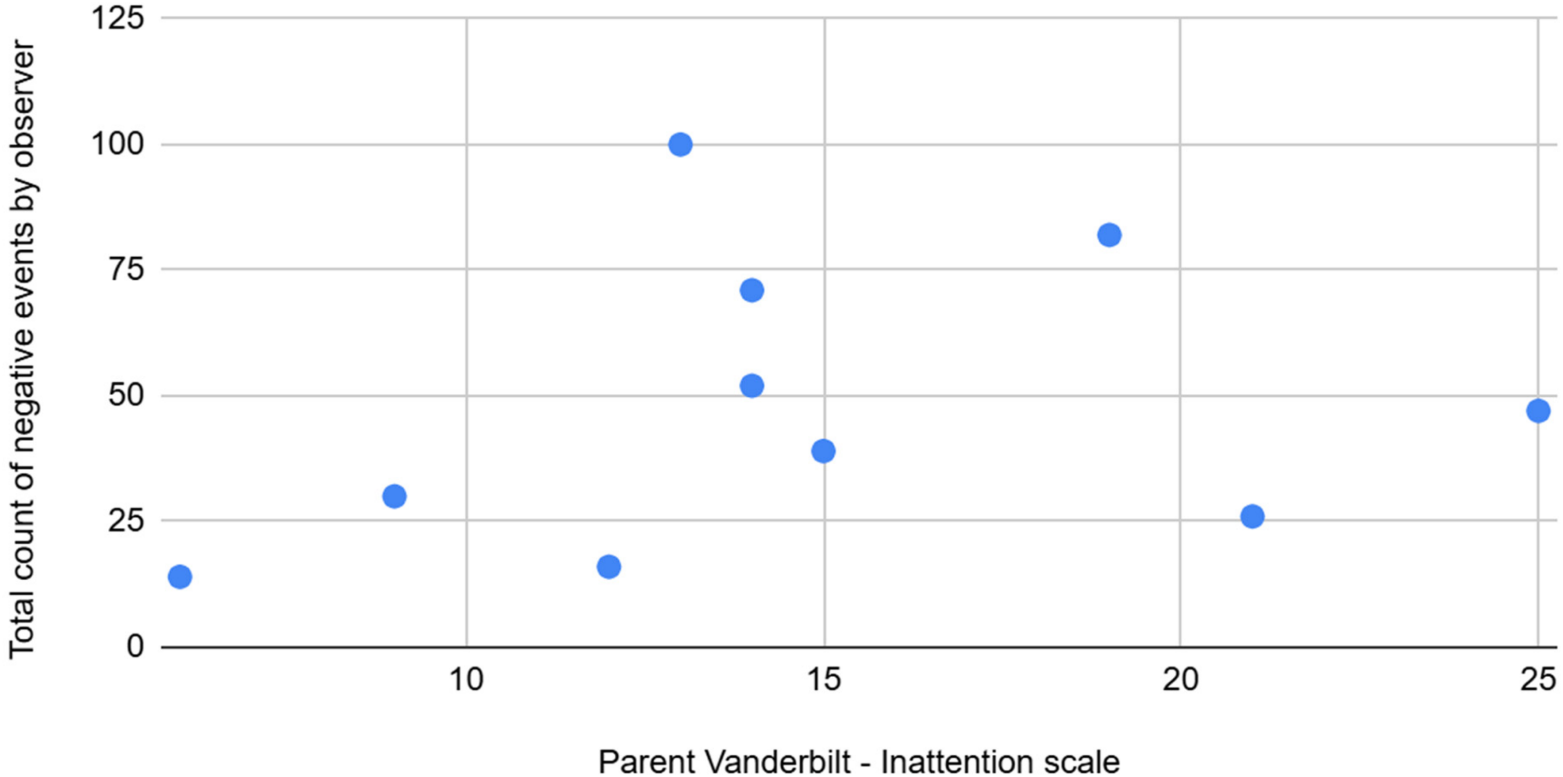
The graph depicting some of the associations between physiological/behavioral variables: Pvanderbilt (l) vs. total count of negative events by observer.

**Figure 5:**
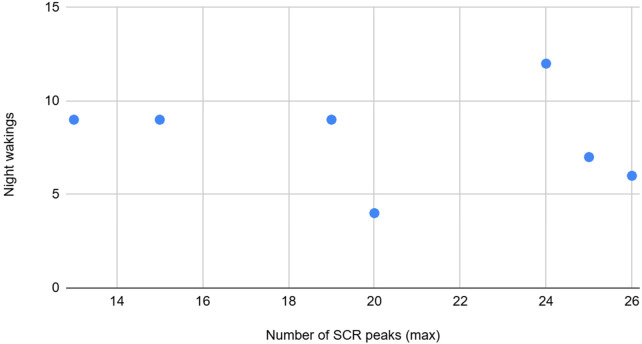
The graph depicting some of the associations between physiological/behavioral variables: SCR peaks (max) vs. night wakings.

**Figure 6:**
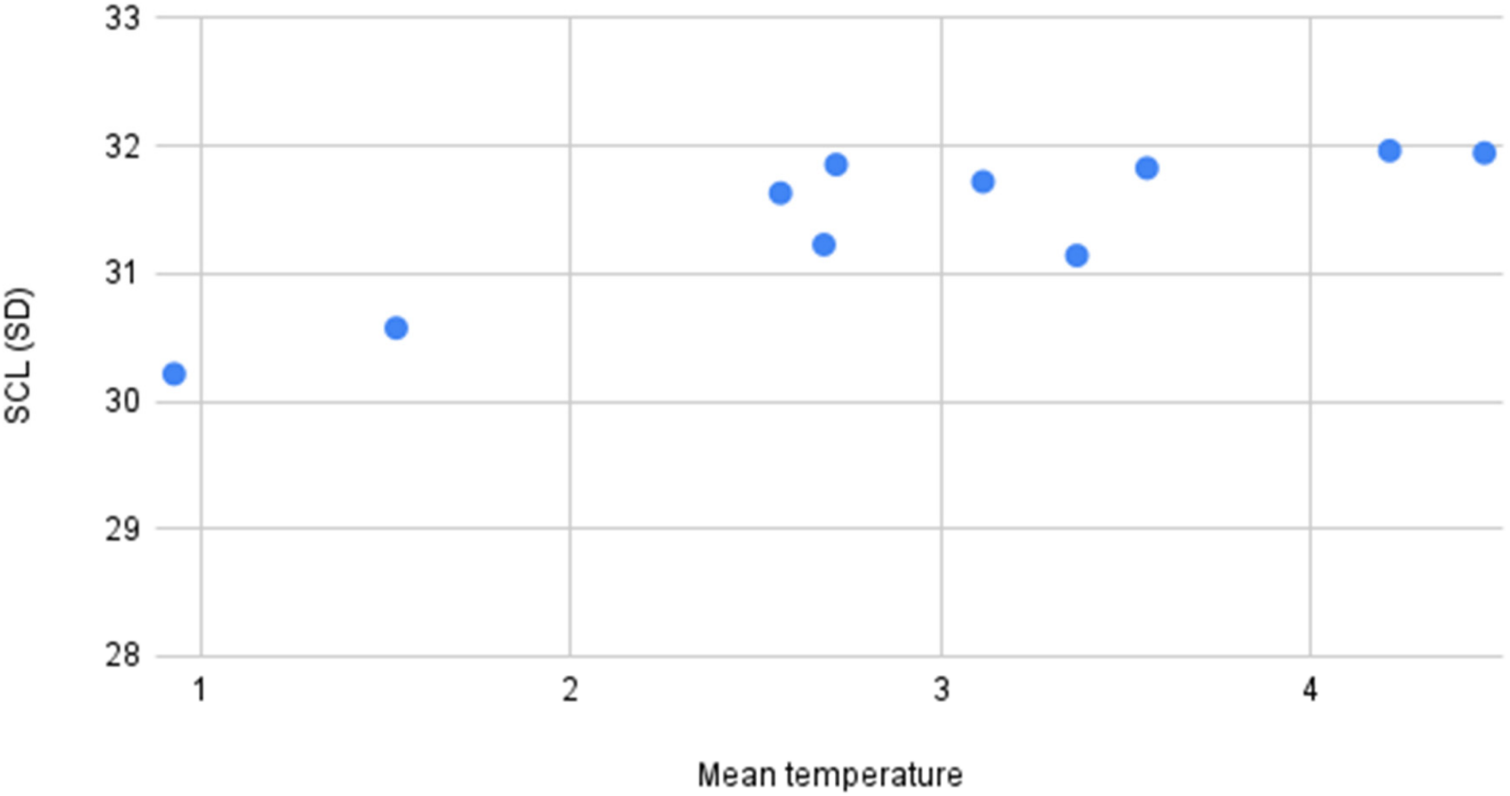
The graph depicting some of the associations between physiological/behavioral variables: Men temperature vs. SCL (SD).

**Figure 7:**
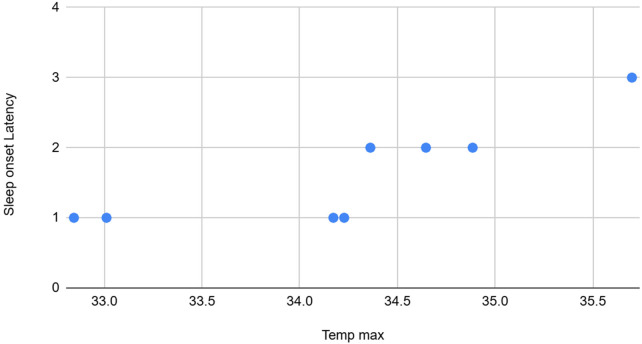
The graph depicting some of the associations between physiological/behavioral variables: Team max vs. sleep onset latency.

**Figure 8:**
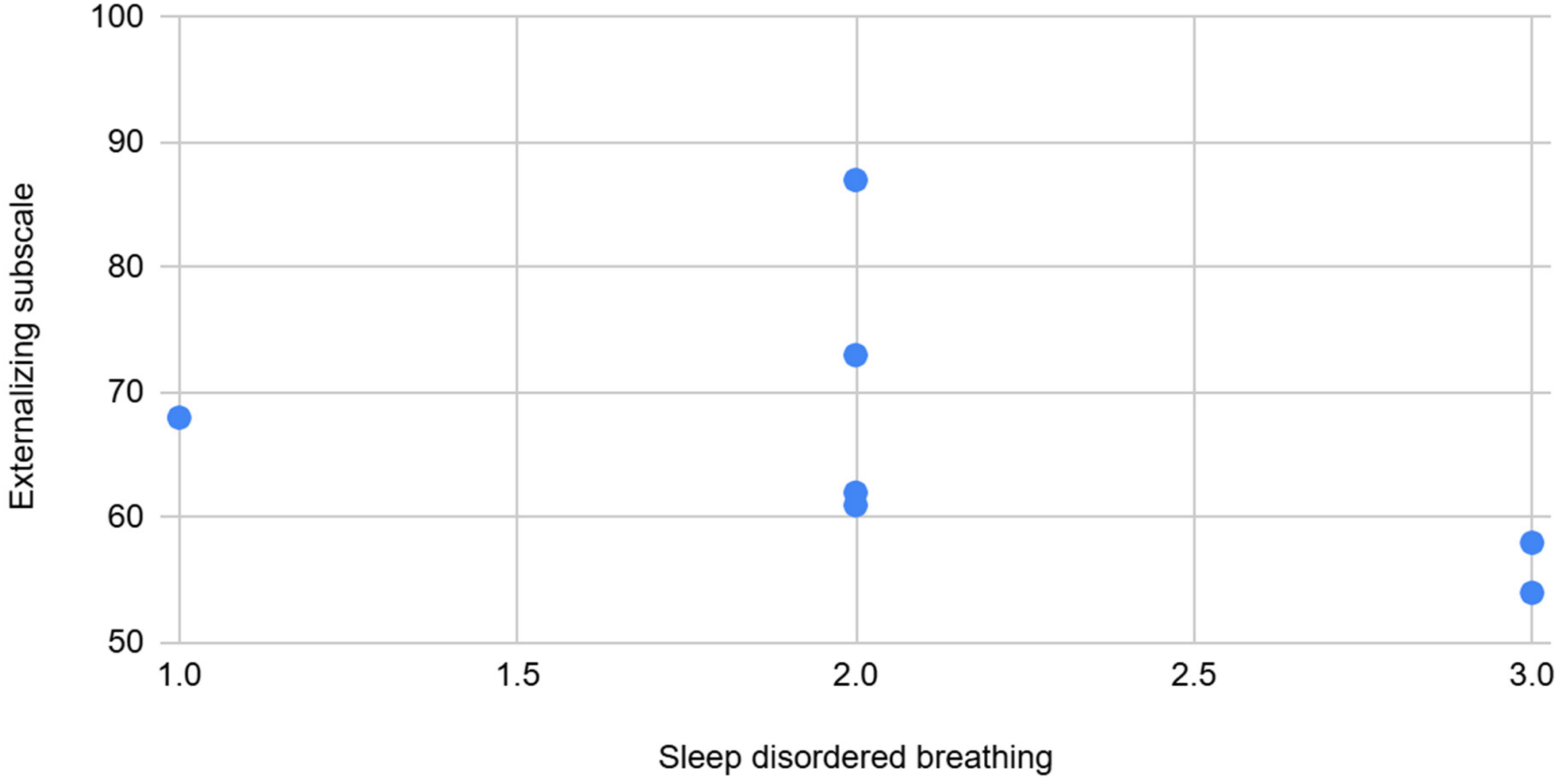
The graph depicting some of the associations between physiological/behavioral variables: Sleep disordered breathing vs. externalizing subscale (BASC).

**Figure 9:**
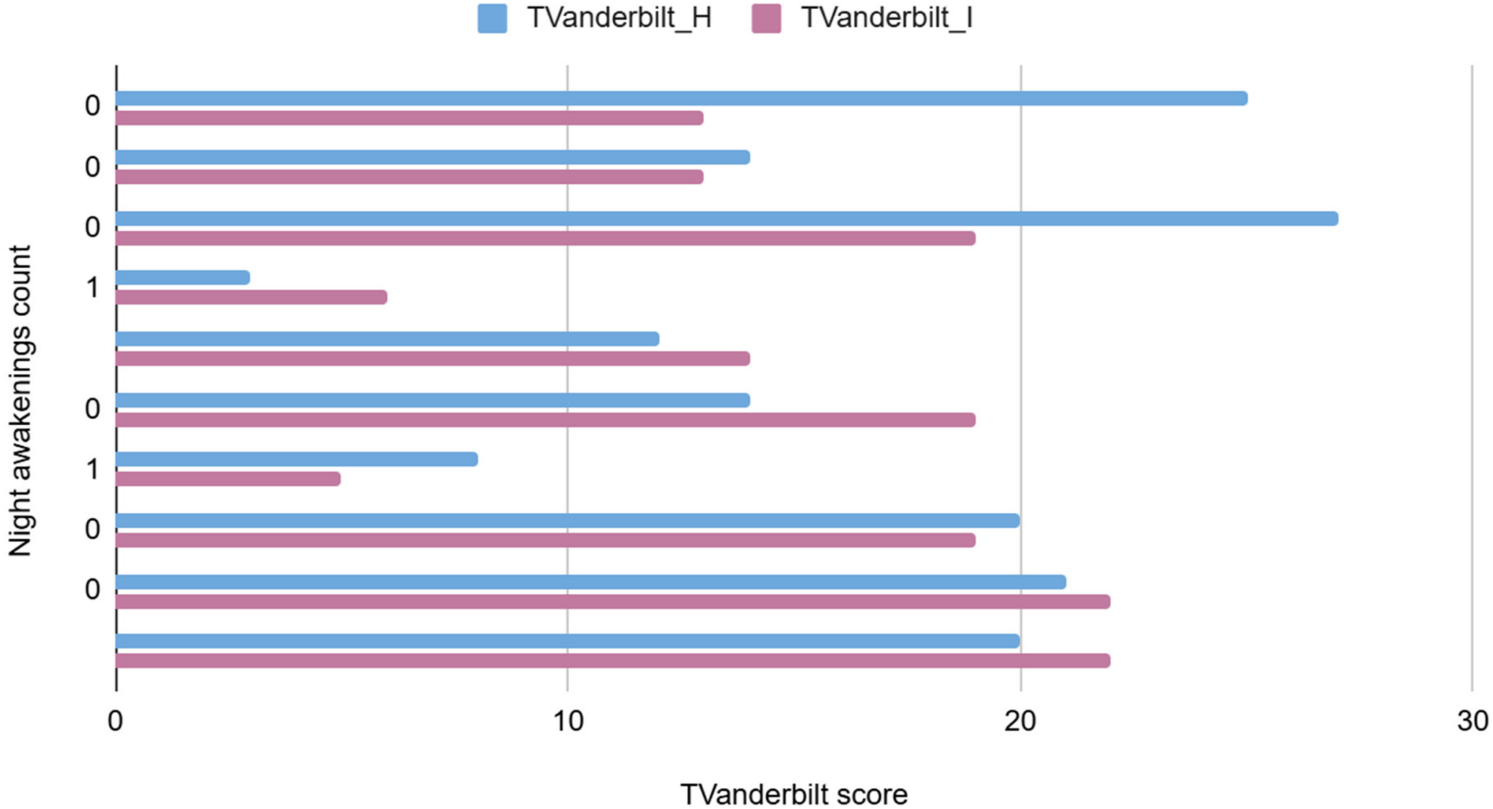
The graph depicting some of the associations between physiological/behavioral variables: Tvanderbilt score vs. night awakenings count.

**Figure 10:**
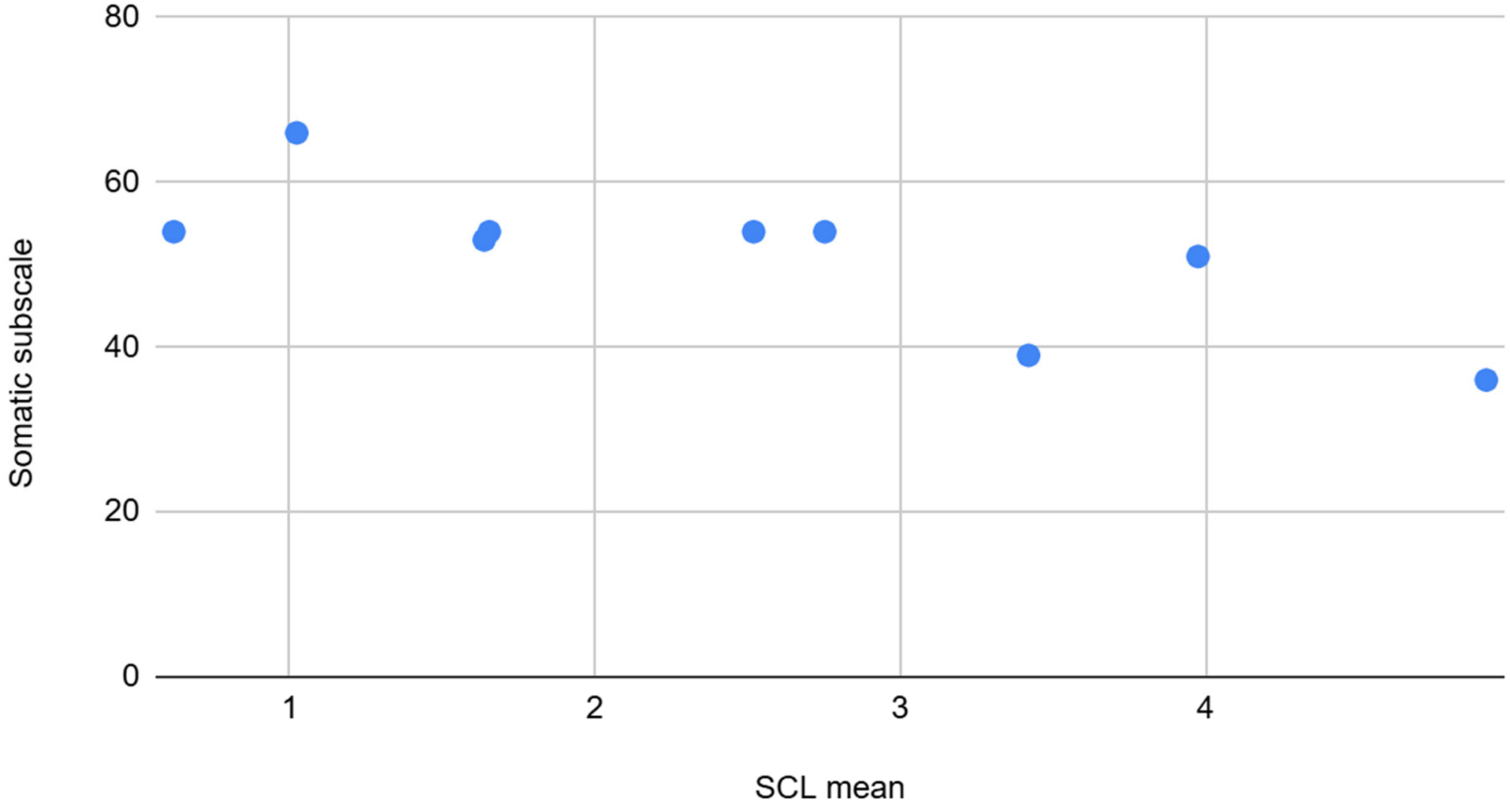
The graph depicting some of the associations between physiological/behavioral variables: SCL mean vs. somatic subscale (BASC).

**Figure 11:**
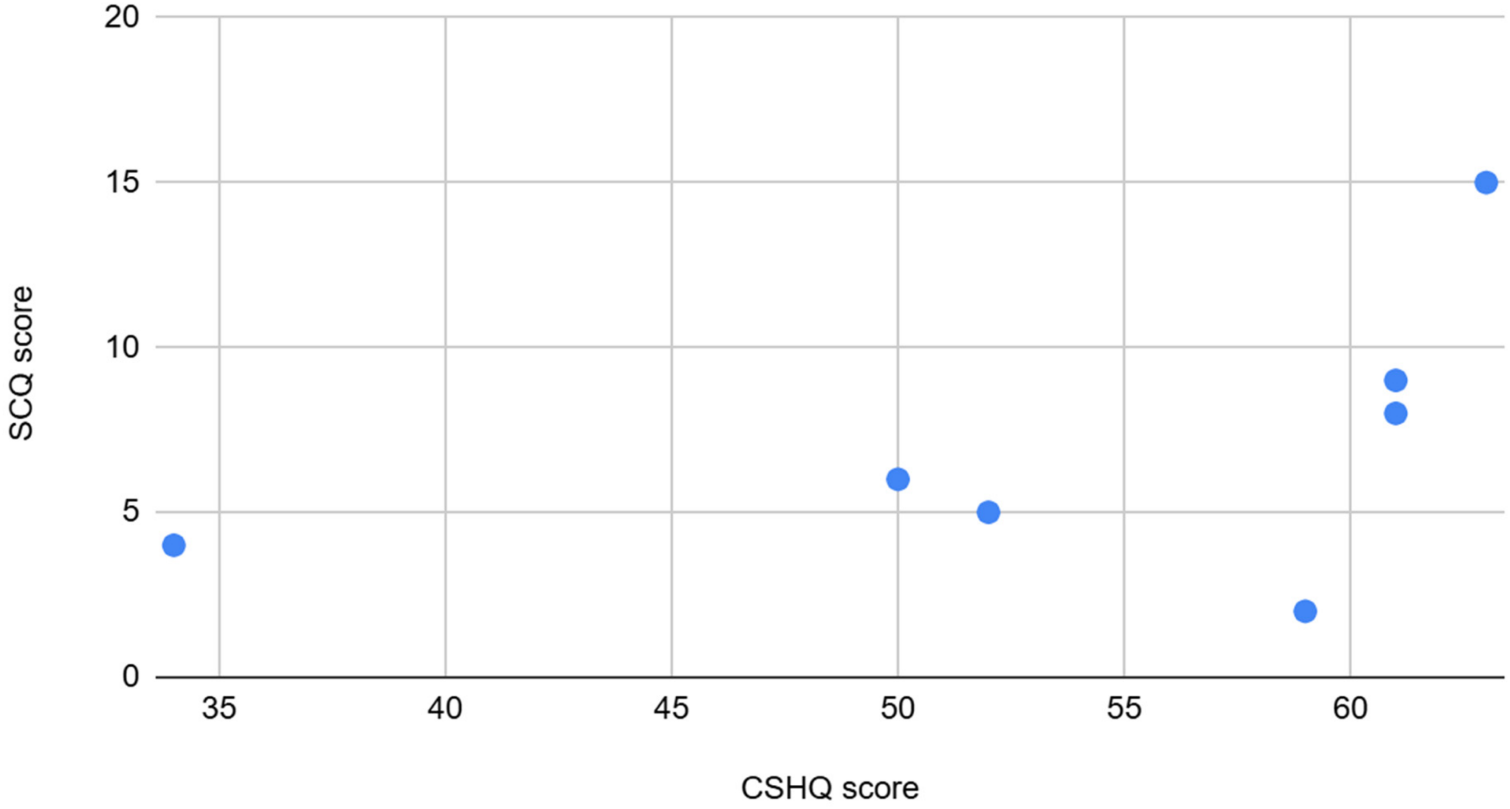
The graph depicting some of the associations between physiological/behavioral variables: CSHQ vs. SCQ score.

EDA features, including the mean, standard deviation, and maximum of the skin conductance response (SCR), exhibited weak positive associations with Behavioral Assessment System for Children (BASC) variables such as hyperactivity, aggression, externalizing behaviors, and anxiety.

The count of night awakenings from the DSF form showed a moderate positive association with the maximum number of SCR peaks from the EDA signal. The standard deviation of the skin conductance level (SCL), a tonic component of EDA, was highly correlated with mean skin temperature. Additionally, the maximum SCL was inversely correlated with the total and average counts of emotional and behavioral outbursts as observed by trained observers.

Associations between EDA features and the Parent/Teacher Vanderbilt scales for hyperactivity and oppositional defiant disorder (ODD) revealed a moderate negative correlation, warranting further investigation. The weak positive association between EDA features (SCR mean, standard deviation, maximum) and BASC variables such as hyperactivity, aggression, externalizing behaviors, and anxiety may reflect the static nature of the BASC assessments, which do not capture the dynamic emotional and physiological states that EDA signals can reveal. A daily behavioral form may provide more accurate insights into the relationship between current child behavior and physiological signals.

Analysis of abnormal event statistics revealed a strong positive association with the parent-reported Vanderbilt scale for inattention, and a weak positive correlation with bed resistance, indicating that increased sleep difficulties are linked to higher abnormal event counts during the day.

Night awakenings and sleep onset delay from the CSHQ forms, along with the total and average counts of abnormal events, showed moderate positive correlations with Skin temperature features (mean and standard deviation). The standard deviation of temperature also had a moderate positive association with the average and total counts of abnormal events recorded by observers, and a weak positive association with the CSHQ score. Conversely, average and total counts of abnormal events observed were moderately negatively correlated with the maximum SCL.

Further analysis of CSHQ subscales revealed that Bed resistance showed a weak positive association with the total/average number of abnormal events. Sleep-disordered breathing exhibited a high negative association with BASC scales for aggression and externalizing behaviors. The CSHQ score and subscales like sleep onset delay, parasomnias, and daytime sleepiness were highly positively associated with the Social Communication Questionnaire (SCQ), indicating that sleep difficulties may correlate with higher SCQ scores, potentially signaling Autism Spectrum Disorders (ASD) and necessitating further evaluation.

Total sleep duration showed a weak negative association with the number of abnormal events, suggesting that children who slept longer experienced fewer abnormal events.

**EDA and Skin temperature physiological signal feature values typically observed in the population** are illustrated in Table 2 below.

3. **Occurrences of emotions observed via Ecological Momentary Assessment (EMA) over all 5 days** have been provided in detail in Table 3 below.

**Table 2:** Relevant features extracted from measured physiological signals (EDA and SKT).

Physiological signal	SCR mean	SCR std	SCR max	SCR peaks mean	SCR peaks max	SCL mean	SCL std	SCL max	Temp mean	Temp std	Temp max
MEAN	0.267	0.481	4.576	5.166	20.3	2.571	2.914	16.636	31.409	1.851	34.101
SD	0.226	0.294	2.215	1.044	4.347	1.355	1.098	8.190	0.609	0.642	0.876

**Table 3:** Occurrences of emotions observed via Ecological Momentary Assessment (EMA) over all 5 days.

EMOTION	Relax	Quiet	Happy	Excited	Laugh	Calm	Yell	Sad	Anxious	Angry	Frustrated	Cry	Negative emotions
MEAN	6.9	18	54.9	17.2	7.3	32.5	5.1	6.5	4	7.2	16.2	8.9	47.9
SD	8.252	26.157	50.350	16.524	11.963	32.813	4.653	5.359	4.830	7.641	10.716	13.74	28.136

These show a mean “abnormal event count” of 47.9 counts as noted via Ecological Momentary Assessment.

**Descriptive statistics from all the scales considered during correlation analysis** have been provided in Table 4 below.

**Table 4:** Descriptive statistics of variables from all the scales considered.

Variables	MEAN	SD	Variables	MEAN	SD
SCR mean	0.267	0.226	Atypical	62.222	9.947
SCR std	0.481	0.294	Withdrawal	66.555	20.706
SCR max	4.576	2.215	BSI	73.4	12.911
SCR peaks number mean	5.166	1.044	Adaptability	38.8	11.631
SCR peaks number max	20.3	4.347	SocialSkills	41.1	9.206
SCL mean	2.571	1.355	Functional Communication	38	9.843
SCL std	2.914	1.098	AdaptiveSkills	37.6	9.143
SCL max	16.636	8.190	Total count of abnormal by observer	47.7	28.817
Temp mean	31.409	0.609	Average count of abnormal by observer	9.54	5.763
Temp std	1.851	0.642	PVanderbilt_I	14.8	5.613
Temp max	34.101	0.876	ISx	4.7	2.830
CSHQ Score	54.285	10.193	PVanderbilt_H	13.8	5.593
Bed resistance	17.857	3.436	Hsx	5.6	4.141
Sleep onset delay	2.428	1.511	PVanderbilt_C	29.6	9.582
Sleep anxiety	4.571	1.902	Csx	9.3	5.598
Sleep duration	2.1428	0.690	PVanderbilt_ODD	7.8	7.192
Parasomnias	6	2.516	ODDsx	2	3.231
Night wakings	8	2.581	PVanderbilt_Anxiety	2.8	2.699
Sleep disordered breathing	2.142	0.690	Anxietysx	0.4	0.699
Daytime sleepiness	11.142	2.609	TVanderbilt_I	15.2	6.106
Nap count	0.375	0.517	Isx	5.1	2.923
Nap duration	0.875	1.356	TVanderbilt_H	16.4	7.560
Bed time	2.125	0.640	sx	5.6	3.0983
Sleep onset latency	1.625	0.744	TVanderbilt_C	30.7	14.651
Night awakenings counts	0.25	0.462	sx	10.5	5.835
Night awakenings duration	0.375	0.744	TVanderbilt_ODD	4.9	3.725
Wake time	1.875	0.353	sx	1.1	0.994
Total sleep duration	3.375	0.744	TVanderbilt_Anxiety	3.3	3.400
BASCHyperactivity	74	7.902	sx	0.6	1.074
Aggression	66.4	16.174	39	3.3	0.483
Externalizing	72	14.460	40	3.8	0.918
Anxiety	56.1	11.308	41	3.4	1.349
Depression	67	17.159	42	3.5	0.849
Somatic	51.2222222	8.899	43	4	0.866
Internalizing	60.5	13.443	SCQ	5.9	3.928
Attention	68.9	3.813			

## Conclusions and discussion

Overall, there were no significant tolerability issues or adverse events associated with wearing the watch despite some minor adjustment issues and the overall palatability appeared to be acceptable.

Initial findings support the potential of biomarker based on real-time physiological monitoring, including photoplethysmography (PPG), electrodermal activity (EDA), skin temperature, and accelerometry, to capture subtle physiological changes that are associated with and may precede emotional outbursts. This study also highlighted the association between daytime behaviors, emotion dysregulation, and sleep duration.

There is a need for reliable and valid measures of emotion dysregulation that can be used in young children at risk for continuing and more severe psychopathology. As demonstrated, a biomarker driven approach has wide potential in both educational and clinical settings to increase understanding of these youth and in the development of effective interventions.

In this study, we observed instances where elevations in electrodermal activity (EDA) were detected without corresponding notations of abnormal behavior in observer records. This discrepancy suggests that EDA, as a physiological marker, may capture subtle fluctuations in emotional arousal that are not overtly observable or consciously recognizable by external observers. Despite the lack of visible behavioral indicators, these physiological changes are significant because they may reflect underlying emotional or psychological states that are not easily detectable through traditional observation methods alone. This is an important highlight for objective biomarker data like EDA in complementing behavioral assessments.

This research not only enhances our understanding of biomarkers associated with emotion dysregulation but also pioneers new strategies for assessment and timely interventions in “real world” settings. Our findings suggest a future where the integration of technology into educational settings enables educators to create more responsive, supportive, and effective learning environments for young children with ADHD, fostering both their emotional and academic development.

## Limitations

There were several limitations to this study, most prominently, this pilot study involved a small sample size. A small sample size in research can lead to several implications, including: reduced reliability of results, increased chance of random variation, difficulty in generalizing findings to a wider population, and a higher risk of drawing incorrect conclusions due to outliers or chance occurrences; essentially, making it harder to confidently interpret the data and potentially undermining the validity of the study. Expanding this pilot study methodology to a larger number of participants would allow for more robust conclusions. The study sample was also relatively heterogeneous in age and presentation, including some who were on medications for emotional dysregulation (specifically guanfacine extended-release, GXR). This heterogeneity within the sample and setting could have introduced confounding factors that affected the observed outcomes, though it is typical of the daily in-class environment. These limitations highlight the need for future studies to address these issues by employing more standardized observational protocols, increasing sample size, and considering the potential confounding effects of medication and prenatal exposure to substances. This was a “real-world” study that was not conducted in a simulated laboratory environment. As such there was a lack of Experimental Stress Induction. As a result, the emotions observed were those that naturally surfaced within a well-controlled, therapeutic environment, without controlled or consistent stress-inducing stimuli. This approach may have led to variability in the types and intensities of emotions captured, potentially limiting the range of data captured across participants. Being a real-world study, there was variability in Observation Timing. Observations were conducted at different times and on different days, leading to variability in the classes, subjects, teachers, observers, and activities participants were exposed to during the study. This variability may have influenced the emotional and physiological responses observed, complicating group comparisons across participants and time points. This, however, is precisely the strategy that will preserve ecological validity of the data and provide personalized algorithms for support of individualized interventions.

Observer Consistency and Human Error. In small observational studies such as these, even tiny increments of variability across observer-raters can be problematic. Despite interrater training and reliability checks, pre-study experiences and education, training levels, and the potential for human error in manual notetaking and timestamping can have notable effects. The use of various emotion tags based on subjective observation may also introduce variability in ratings. This is another notable reason for the often-discrepant findings between real-world and simulated classroom lab research. In our study, we opted for real-world data.

## Conclusion

This pilot, observational study with high-risk young children with ADHD and emotion dysregulation attending a special education school placement examined the feasibility and reliability of collecting physiological signal data from biosensors, and comparing this data with observer notes, additional sleep and behavioral information. The study successfully established the viability of monitoring physiological fluctuations in emotionally dysregulated youth with ADHD. The data derived from this analysis was instrumental in developing an algorithm, which will be used to train and build a machine learning model. Importantly, this study demonstrated that Ecological Momentary Assessment using a digital device is both feasible and tolerable for this population in a school setting, warranting further research examining the predictive validity and clinical utility.

It is important to note that the selection and combination of physiological signals may impact the prediction of emotional states and dysregulation. Similarly, the choice of parameters extracted from these signals can influence the accuracy of predictions. We plan to replicate this study with a larger population to further validate our findings. The data collected will contribute to the development of a proprietary machine learning-driven pediatric digital health system. Predicting emotions and identifying impending meltdowns will potentially assist parents, caregivers, teachers, and clinicians in managing stress and problematic behaviors before they escalate, supporting Just-In-Time Adaptive Interventions (JITAI) throughout daily life.

## Data Availability

The datasets presented in this article are not readily available because the data is being held for a larger project for expanded sample size and development of proprietary algorithms. Requests to access the datasets should be directed to Ripudaman Singh, zeeba.singh@maxishealth.com.

## Footnotes

^1^Biosensor wearable: Empatica Embrace Plus wearable (https://www.empatica.com/embraceplus/).
