# Supplementary material for: Ecological Momentary Assessment of emotional dysregulation and outbursts among youth with ADHD: a feasibility study of a biomarker-driven predictive algorithm in the special education pre-K and early childhood classroom settings
Source: Front Child Adolesc Psychiatry. 2025 Mar 21;4:1549220. doi: 10.3389/frcha.2025.1549220 (PMC11970134; doi:10.3389/frcha.2025.1549220)
Supplement: Supplementary file 1 [file Datasheet1.docx]

**Supplementary Information**

DAILY SLEEP FORM

**Participant ID: _______________**

**Parent’s note:** the form below is to be **ONLY** filled in by a **Parent / caregiver** of the child (subject) inducted in our study. It is to be filled in with utmost accuracy and without any bias. This form is to be filled in **each morning** – based on the **sleep their child had last night**. This form is to be handed over to the child before he/she goes to school each day. The child will have to submit this form to our Study observer/ PI on site/ in the classroom.

**Date: _____________                                                                             Parent signature:**

| **Q no.** | **Question** | **Response** |
| --- | --- | --- |
| 1 | How many times did your child take a nap in the day? | 0  1 – 2 times  3 – 4 times  5 or more times |
| 2 | How long did your child nap in the day? | Not at all  Less than 30 minutes  30 minutes to an hour  More than an hour |
| 3 | At what time did your child go to bed last night? | Before 7pm  8pm to 10 pm  10pm to 12am  After 12 am |
| 4 | After settling down, how long did it take for your child to fall asleep? | Within 20 minutes  20 to 45 minutes  45 minutes to an hour  More than 1 hour |
| 5 | After falling asleep, how many times did your child wake up at night? | Not at all  1 -2 times  3 - 4 times  5 or more times |
| 6 | What was the reason for your child waking up at night? | Illness / Medical condition  Hunger  Stress / Anxiety  Nightmares / Night terrors  Did not wake up |
| 7 | After falling asleep, for how long was your child awake during the night in total? | Less than 15 minutes  30 minutes to an hour  More than an hour  Did not wake up |
| 8 | At what time did your child wake up in the morning? | Before 6 am  6 to 8 am  8 to 10 am  After 10 am |
| 9 | How many hours did your child sleep for last night? (including any awakenings at night) | 1 – 4 hours  4 – 6 hours  6 – 8 hours  More than 8 hours |
| 10 | In what setting did your child sleep last night? | Co-sleep in the same room  Co-sleep on the same bed  They came to your room in the middle of the night  They slept through the night in a different room |

Thank you
